# A filter-based feature selection approach for identifying potential biomarkers for lung cancer

**DOI:** 10.1186/2043-9113-1-11

**Published:** 2011-03-21

**Authors:** In-Hee Lee, Gerald H Lushington, Mahesh Visvanathan

**Affiliations:** 1 Bioinformatics Core Facility, University of Kansas, Lawrence, KS 66046, USA

## Abstract

**Background:**

Lung cancer is the leading cause of death from cancer in the world and its treatment is dependant on the type and stage of cancer detected in the patient. Molecular biomarkers that can characterize the cancer phenotype are thus a key tool in planning a therapeutic response. A common protocol for identifying such biomarkers is to employ genomic microarray analysis to find genes that show differential expression according to disease state or type. Data-mining techniques such as feature selection are often used to isolate, from among a large manifold of genes with differential expression, those specific genes whose differential expression patterns are of optimal value in phenotypic differentiation. One such technique, Biomarker Identifier (BMI), has been developed to identify features with the ability to distinguish between two data groups of interest, which is thus highly applicable for such studies.

**Results:**

Microarray data with validated genes was used to evaluate the utility of BMI in identifying markers for lung cancer. This data set contains a set of 129 gene expression profiles from large-airway epithelial cells (60 samples from smokers with lung cancer and 69 from smokers without lung cancer) and 7 genes from this data have been confirmed to be differentially expressed by quantitative PCR. Using this data set, BMI was compared with various well-known feature selection methods and was found to be more successful than other methods in finding useful genes to classify cancerous samples. Also it is evident that genes selected by BMI (given the same number of genes and classification algorithms) showed better discriminative power than those from the original study. After pathway analysis on the selected genes by BMI, we have been able to correlate the selected genes with well-known cancer-related pathways.

**Conclusions:**

Our results show that BMI can be used to analyze microarray data and to find useful genes for classifying samples. Pathway analysis suggests that BMI is successful in identifying biomarker-quality cancer-related genes from the data.

## Background

Lung cancer accounts for large portion of cancer deaths (29%) in the United States for men as well as woman [1]. The major types of lung cancer are small-cell and non-small-cell cancer. Non-small-cell cancer can be further divided into three histological subtypes: squamous-cell carcinoma, adenocarcinoma and large cell lung cancer [2]. Regardless of subtype, the 5-year survival rate for lung cancer is among the lowest of all cancers at 15% (data for USA) [1]. Since the treatment of lung cancer depends on the subtype and the stage of cancer, it is important to have determined specific molecular biomarkers that can identify the type of cancer as a function of genes closely related to each distinct phenotype.

With advance of microarray technologies, it is possible to conduct high throughput determination of the relative rates with which genes are expressed in a given cell or tissue type. This can help researchers better understand a disease at the genomic level and has become an important tool in biological sciences as well as medical and pharmaceutical research. In the context of lung cancer, microarray technology can be used to identify genes whose expression profile in a type of cancer differs from normal tissues or from other types of cancer. Such biomarkers are important since they can provide the basis for improving a diagnostic classifier or for enhancing the prediction of patient-specific prognosis or therapeutic response [3]. From an informatics perspective, the process of selecting differentially expressed genes is readily achieved via data-mining techniques known as feature selection. Feature selection, an important step in the data-mining process, aims to find representative feature subsets that meet desired criteria. In microarray data analysis, one criterion for a desired feature subset would be a set of genes whose expression patterns vary significantly when compared across different sample groups. The resulting subset can then be used to further analysis such as building a diagnostic classifier.

Feature selection methods, in general, can be categorized into three types, depending on how they are combined with other analysis steps: filter methods, wrapper methods and embedded methods [4]. Filter methods assess the relevance of features as scores by looking only at the properties of the data. Features can be sorted by their scores and low-scoring features can be removed. Wrapper methods embed the analysis model within the feature subset search. In this setup, a subset of features is evaluated by applying a specific analysis model to reduced data with the selected feature subset. In embedded methods, the search for an optimal feature subset is built into the analysis algorithm. Filter methods are the most commonly applied in bioinformatics studies since they are computationally simple, fast and independent of other analysis algorithms. Also they allow features to be quantified and prioritized according to the scores, which is particularly important for biological interpretation.

In this paper, a filter-based feature selection method, biomarker identifier (BMI), is adopted to analyze gene expression data that might be used to discriminate between samples with and without lung cancer. The data consists of gene expression patterns in histologically normal large-airway epithelial cells obtained via bronchoscopy from smokers. Genes identified using this data set can be used to diagnosing lung cancer among smokers with suspected lung cancer. The genes selected by BMI were compared with those from various other feature selection algorithms and those identified from the original experimental study. Pathway analysis for the genes selected by BMI was also performed.

## Methods

### Biomarker Identifier

The biomarker identifier (BMI) [5,6] method combines various statistical measures to discern the ability of features to distinguish between two data groups of interest. It considers three measures for evaluating features. First, it checks whether distribution of a feature is significantly different between data groups. If the distribution of a feature changes substantially, the feature might be relevant to the underlying difference between data groups. Second, the ratio of overall variance relative to variance in control group is used to measure the reliability of a feature. For example, if the overall variance is greater than that of control group, it means that the feature displays more noisy behavior in experiment group making it less useful unless it also demonstrates a significant change between data group. On the other hand, an overall variance smaller than that of control group implies that the feature shows more consistent behavior in the experiment group, making it a more useful feature provided that there exists a significant difference between the contrasted data groups. For these reasons, BMI penalizes or credits a score of a feature by the ratio of overall variance relative to variance in control group. Lastly, BMI considers the discriminative power of each individual feature by incorporating the true positive rate from logistic regression using the feature. In mathematical terms, let us assume a data set *D *consisting of two groups 'control (ctr)' and 'experiment (exp)'. BMI assigns a score for a feature *x *defined as follows:

where

Here, λ is a scaling factor and *TP*^2 ^is the product of the true positive (TP) rates determined for each groups using logistic regression of the form 'outcome ~ feature'. *CV_ctr _*and *CV *denote the coefficient of variance for the feature *x *in the 'control' group and in both groups, respectively. Also, Δ = , where , and  denote the mean value of *x *in 'control' and in both groups, respectively. For biological data such as microarray, the sign of Δ*_diff _*for a particular gene can be interpreted as over-expression or under-expression in 'experiment' compared to 'control'; positive as over-expression and negative as under-expression.

BMI has shown promising results on various data sets such as mass spectrometry data of metabolites [5], liver disease [7] and microarray data from various types of cancer [6]. In this study, it is used to identify potential biomarkers for lung cancer from microarray data.

### Other feature selection methods

For comparison with BMI, we used 6 different popular feature selection methods: information gain (IG), Relief-F (RF), t-test (T) and its two variants (moderated t-test (MT) and window t-test (WT)), and chi-squared test (CS).

#### Information gain

Information gain (relative entropy, or Kullback-Leibler divergence), in probability theory and information theory, is a measure of the difference between two probability distributions. It evaluates a feature *x *by measuring the amount of information gained with respect to the class (or group) variable *y*, defined as follows:

Specifically, it measures the difference between the marginal distribution of observable *y *assuming that it is independent of feature *x *(*P*(*y*)) and the conditional distribution of *y *assuming that it is dependent of *x *(*P*(*y*|*x*)). If *x *is not differentially expressed, *y *will be independent of *x*, thus *x *will have small information gain value, and vice versa.

#### Relief-F

Relief-F [8] is an instance-based feature selection method which evaluates a feature by how well its value distinguishes samples that are from different groups but are similar to each other. For each feature *x*, Relief-F selects a random sample and *k *of its nearest neighbors from the same class and each of different classes. Then *x *is scored as the sum of weighted differences in different classes and the same class. If *x *is differentially expressed, it will show greater differences for samples from different classes, thus it will receive higher score (or vice versa).

#### t-test and variants

The Student's t-test [9] is traditionally used to compare two normally distributed samples or populations. It prefers features with a maximal difference of mean value between groups and a minimal variability within each group, but it can fail when there are small number of samples or the estimated variances are not equal between groups (heteroscedasticity): scenarios which are common for practical data. To cope with such problems, Welch proposed a variant of t-test taking heteroscedasticity into account [10]. Various statistical tests for differential expression are based on the traditional Student and Welch tests. Smyth [11] applied a hierarchical Bayesian approach (moderated t-test) to the Student and Welch tests and integrated more *a priori *information to yield more robust estimates. Berger *et al. *[12] suggested a window t-test that uses multiple genes which share a similar expression level to compute the variance to be incorporated in the t-test. In this work, we chose Welch's t-test, moderated t-test and window t-test for comparison.

#### chi-squared test

Chi-squared test is another popular statistical test of the divergence between the observed and expected distribution of a feature. In feature selection, it tests whether the distribution of a feature differs between groups. The chi-square score uses the summation of squared differences between observed and expected values divided by expected values.

### Experimental data

Spira *et al. *reported gene expression data from large airway epithelial cells by microarray analysis [13]. This data set covers a set of 129 Affymetrix HG-U133A microarrays comparing 60 smokers with lung cancer and 69 smokers without lung cancer. This experiment was designed to determine if gene expression in histologically normal large-airway epithelial cells obtained via bronchoscopy from smokers with suspected lung cancer could be used as a lung cancer biomarker. In this data set, 7 genes were confirmed to be differentially expressed between cancerous samples and non-cancerous samples by quantitative PCR [13]. The Robust Multichip Average (RMA) algorithm [14] was used for background adjustment, normalization, and probe-level summarization of the microarray samples (please refer to supplementary methods of [13] for detailed information). The data set can be accessed from gene expression omnibus (GEO, http://www.ncbi.nlm.nih.gov/geo/) under accession number of GSE4115. This data set was chosen since it consisted of a significant number of replicates and some of the genes in the data set were confirmed by quantitative PCR, which provides a good basis for preliminary validation.

To contrast performance among feature selection methods, we also used the dataset published through MicroArray Quality Control project phase II (MAQC-II). Among 9 non-control data sets from MAQC-II, the data set with the most balanced number of positive/negative samples (breast cancer data with estrogen receptor status as class) was chosen. The data set consists of training (130 samples) and validation (100 samples) sets. The processed data was obtained through GEO under accession number GSE20194.

## Results and Discussion

### Comparison with other feature selection methods

Feature selection methods can be evaluated in various ways. One popular way is to observe the classification performance using the features selected by the method. If a feature selection method is able to choose truly significant features, the classifier trained using those features should show good performance with a small number of features. If important features are already known, on the other hand, we can evaluate feature selection methods by how they rank those known features. Since important features have not been reported for the MAQC-II data set, it can be approached only via the first evaluation strategy, but the airway data set is amenable to both modes of evaluation since some of genes have been experimentally confirmed to be differentially expressed.

Since a separate validation set is available within the MAQC-II data, we used the training set for feature selection and validation set for classification. That is, feature selection methods are first applied to training set to obtain feature subsets. Then, for each feature selection method/classification algorithm pairing, classification performances are evaluated on the validation set through 10-fold cross-validation with varying number of features (from 1 to 60). AUC values (area under the curve; a popular measure for model comparison in machine learning research interpreted as the probability that, given a randomly picked positive example and negative example, the classifier will assign a higher score to the positive example than to the negative one) have been used herein to measure classification performance. Larger AUC values imply more precise classification. For implementation, we used Weka [15], a popular machine learning library written in Java, and the default setting was used for each classification algorithm. Table 1 shows the maximum AUC value achieved by each combination of feature selection methods and classification algorithms for the MAQC-II data set. We can see that the classifiers in combination with BMI show performance levels comparable to others with relatively small number of features. Also, the features selected by BMI show stable performance regardless the classification algorithm.

**Table 1:** Comparison of classification performances on MAQC-II data set

	Classification Algorithms			
**Feature Selection Methods**	**Support Vector Machine**	***k*-Nearest Neighbor**	**Naive Bayes**	**Random Forest**

Information Gain	0.9031 (6)	0.9380 (25)	0.9008 (40)	0.9206 (50)
Chi-squared test	0.8821 (1)	0.9164 (50)	0.9151 (4)	0.9441 (60)
Relief-F	0.8821 (1)	0.9052 (15)	0.8995 (50)	0.9306 (60)
t-test	0.9067 (15)	0.9100 (20)	0.9042 (8)	0.9304 (40)
Window t-test	0.8903 (5)	0.9216 (5)	0.9012 (2)	0.9199 (10)
Moderated t-test	0.8903 (6)	0.9084 (5)	0.8987 (1)	0.9309 (50)
BMI	0.9077 (4)	0.9298 (15)	0.9164 (4)	0.9250 (9)

Each value represents the maximum AUC value (by 10-fold cross-validation) achieved by the corresponding feature selection method and classification algorithm. The number of features used to achieve the maximum is shown inside parenthesis.

For the airway data set, we applied a similar ten-fold cross-validation approach as with the MAQC-II data to compare classification performance of different feature selection methods. Here, the data was divided into 10-folds, whereby 9 folds are used for both selecting features and training classifiers, and the reserved fold was used to calculate AUC value of trained classifiers. For each combination of feature selection methods and classification algorithms, this process was repeated 10 times with a different reserved fold, while varying number of features (from 1 to 60) and the AUC values were averaged over the ten distinct reserved-fold cases. The parameter setting for each classification algorithm was the same as in MAQC-II data set. Table 2 shows the maximum AUC value achieved by each combination of feature selection methods and classification algorithms. As in MAQC-II data set, the classifiers in combination with BMI show comparable performance with others with relatively small number of features. And the features selected by BMI show stable performance regardless the classification algorithm.

**Table 2:** Comparison of classification performances on airway data set

	Classification Algorithms			
**Feature Selection Methods**	**Support Vector Machine**	***k*-Nearest Neighbor**	**Naive Bayes**	**Random Forest**

Information Gain	0.6853 (40)	0.8006 (4)	0.8297 (50)	0.8620 (60)
Chi-squared test	0.7052 (20)	0.8029 (60)	0.7997 (3)	0.8309 (50)
Relief-F	0.6633 (25)	0.7825 (9)	0.8329 (25)	0.8685 (60)
t-test	0.6902 (8)	0.7822 (4)	0.8402 (4)	0.8121 (8)
Window t-test	0.6856 (20)	0.7817 (30)	0.8367 (20)	0.8093 (40)
Moderated t-test	0.6878 (6)	0.7875 (5)	0.8329 (5)	0.8115 (20)
BMI	0.7572 (9)	0.8005 (5)	0.8299 (5)	0.8212 (10)

Each value represents the maximum AUC value (via 10-fold cross-validation) achieved by the corresponding feature selection method and classification algorithm. The number of features used to achieve the maximum is shown inside parenthesis.

**Figure 1:**
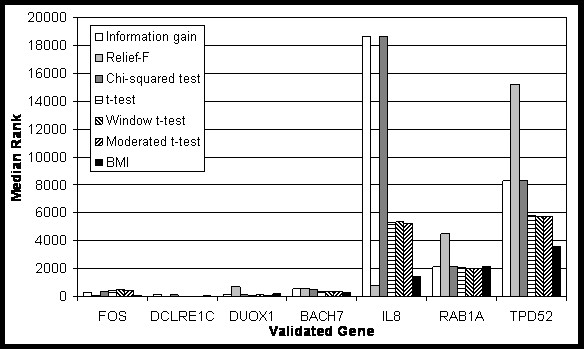
**The median ranks of validated genes in airway data set by various feature selection methods**.

Next, for the airway data set, we investigated how the genes confirmed in the literature (DUOX1, BACH2, DCLRE1C, RAB1A, TPD52, FOS, and IL8) are ranked by BMI compared to other feature selection methods. If these genes are generally ranked highly, a feature selection method could be said to corroborate the given data. As before, we divided the data into 10 folds and used only 9 folds in feature selection, repeating the feature selection for each distinct reserved fold. For each of these ten fold cases, we recorded gene ranks as determined by each method and calculated the median value for each gene. Figure 1 shows median ranks of validated genes by different feature selection methods, demonstrating that BMI ranks all of the confirmed genes within the top 4000 ranked genes, and the overall BMI ranking of confirmed genes is generally superior to other methods.

From these results, it can be said that BMI shows competitive performance in identifying useful features for classification and shows high consistency with actual differential expression.

### Comparison with biomarkers from literature

For the airway data set, we further compared the genes selected by BMI and the biomarkers from original literature [13]. In original literature, 80 features were selected to distinguish cancerous samples from normal samples. For BMI, we chose 10 features that were used to achieve the best classification performance in Table 2. The selected 10 features are shown in Table 3. Then we trained various popular classification algorithms using these two sets of features: naïve Bayes, support vector machine (SVM), neural network, k-nearest neighbor, and random forest. We used the implementation in Weka software [15] with default settings.

**Table 3:** Top 10 genes selected by BMI

Probe ID	Symbol	Regulation	Name
201694_s _at	EGR1	Up	early growth response 1
202056_at	KPNA1	Up	karyopherin alpha 1 (importin alpha 5)
203265_s_at	MAP2K4	Up	mitogen-activated protein kinase kinase 4
207283_at	RPL23AP13	Down	ribosomal protein L23a pseudogene 13
211612_s_at	IL13RA1	Up	interleukin 13 receptor, alpha 1
214261_s_at	ADH6	Up	alcohol dehydrogenase 6 (class V)
216609_at	TXN	Down	Full length insert cDNA clone YI46D09
219233_s_at	GSDMB	Down	gasdermin B
222339_x_at	-	Down	-
34206 at	ARAP1	Down	ArfGAP with RhoGAP domain, ankyrin repeat and PH domain 1

Table 4 shows the detailed classification performances obtained from 20 independent runs of 10-fold cross-validation. Classifiers trained using features selected by BMI generally showed better performance for most classification algorithms. This implies that the features selected by BMI are more useful for constructing accurate classifiers, which can provide a good basis for further screening of biomarkers.

**Table 4:** Classification performances with selected biomarkers by BMI and original literature

	Biomarkers by BMI			Biomarkers from original literature		
**Classifier**	**Specificity**	**Sensitivity**	**Accuracy**	**Specificity**	**Sensitivity**	**Accuracy**

Naïve Bayes	0.7938++	0.7006++	0.7489++	0.7117	0.6644	0.6872
SVM	0.8134++	0.7056++	0.7615++	0.6622	0.6593	0.6607
Neural Network	0.7242++	0.6422	0.6848	0.6956	0.7459++	0.7217++
k-Nearest Neighbor	0.8325++	0.6144	0.7275++	0.6378	0.6964++	0.6682
Random Forest	0.7139++	0.7328++	0.7230++	0.6872	0.6680	0.6773

++ and + denotes superior performance as determined at of 1% and 5% significance levels respectively.

### Pathway analysis of selected biomarkers

Although a set of genes is useful for training classifier, the constituent genes may be useless as biomarkers if their biological roles are not related to the target disease or process. Thus we analyzed the pathways associated with 80 highly-ranked genes to investigate their biological roles. For pathway analysis, we investigated associated terms in KEGG pathways [16], NCI-Nature pathway interaction database [17], and PANTHER (protein analysis through evolutionary relationships) classification system [18] using the EGAN program [19].

Tables 5 and 6 summarize the genes and their associated pathways with significant *p*-values (< 0.05). We can observe that there are some genes (EGR1, FOS, DUSP10, and MAP2K4) associated with mitogen-activated protein kinase (MAPK) pathways, which is a well-known target in the oncology drug discovery [20]. Also, three genes (APC, MSH2, and ATF3) showed significant association with a term from the NCI-Nature Pathway Interaction Database, 'Direct p53 effectors.' This implies that those genes are related with protein 'p53' which is known as a tumor suppressor protein [21]. We note that incidence of the general KEGG annotation 'pathways in cancer' showed a good association (*p*-value of 0.0019) with our set of 80 genes. One also finds other pathways related with known oncogenes such as c-Met [22] and epidermal growth factor receptor (EGFR or ErbB-1) [23] within our list. From these, it can be said that genes highly ranked by BMI are generally relevant to cancer development or diagnosis, thus BMI appears to be useful for identifying potential biomarkers for lung cancer.

**Table 5:** KEGG pathways and PANTHER classifications associated with top 80 genes selected by BMI

KEGG pathway name	*p*-value	Associated genes
Colorectal cancer	1.3809E-4	FOS, MSH2, APC
Pathways in cancer	0.0019	FOS, MSH2, APC, TCEB2
Metabolic pathways	0.0021	ADH6, SAT1, EXT2, TGDS, BTD, PRPS1, AGPS
Biotin metabolism	0.0032	BTD
MAPK signaling pathway	0.0094	DUSP10, MAP2K4, FOS
Cytokine-cytokine receptor interaction	0.0098	CXCR4, ACVR2A, IL13RA1
Toll-like receptor signaling pathway	0.0117	FOS, MAP2K4
Tight junction	0.0196	PPP2R2 D, INADL
Mismatch repair	0.0361	MSH2
Glycosaminoglycan biosynthesis - heparan sulfate	0.0408	EXT2
Pentose phosphate pathway	0.0423	PRPS1
Endocytosis	0.0428	ARAP1, CXCR4

PANTHER classification	*p*-value	Associated genes

Oxidative stress response	8.6417E-5	TXN, MAP2K4, DUSP10
O-antigen biosynthesis	0.0064	TGDS
T cell activation	0.0083	FOS, B2M
Interleukin signaling pathway	0.0108	IL13RA1, FOS
Apoptosis signaling pathway	0.0133	ATF3, FOS
FGF signaling pathway	0.0135	MAP2K4, PPP2R2D
Axon guidance mediated by Slit/Robo	0.0253	CXCR4
Hypoxia response via HIF activation	0.0408	TXN
Insulin/IGF pathway-mitogen activated protein kinase kinase/MAP kinase cascade	0.0484	FOS

**Table 6:** NCI-Nature pathway interactions associated with top 80 genes selected by BMI

NCI-Nature Pathway Interaction	*p*-value	Associated genes
ATF-2 transcription factor network	6.8276E-5	ATF3, FOS, DUSP10
Downstream signaling in naïve CD8+ T cells	1.8173E-4	B2 M, EGR1, FOS
Signaling events mediated by Hepatocyte Growth Factor Receptor (c-Met)	2.6255E-4	EGR1, MAP2K4, APC
Ephrin B reverse signaling	8.6116E-4	CXCR4, MAP2K4
ErbB1 downstream signaling	8.7013E-4	MAP2K4, FOS, EGR1
Regulation of p38-alpha and p38-beta	0.0011	DUSP10, MAP2K4
Direct p53 effectors	0.0013	APC, MSH2, ATF3
Trk receptor signaling mediated by the MAPK pathway	0.0014	EGR1, FOS
RhoA signaling pathway	0.0021	FOS, MAP2K4
IL6-mediated signaling events	0.0023	MAP2K4, FOS
Presenilin action in Notch and Wnt signaling	0.0024	FOS, APC
Calcineurin-regulated NFAT-dependent transcription in lymphocytes	0.0025	EGR1, FOS
Regulation of Androgen receptor activity	0.0027	EGR1, MAP2K4
Fc-epsilon receptor I signaling in mast cells	0.0041	FOS, MAP2K4
IL12-mediated signaling events	0.0045	B2 M, FOS
HIF-1-alpha transcription factor network	0.0052	FOS, CXCR4
CDC42 signaling events	0.0058	APC, MAP2K4
Regulation of nuclear SMAD2/3 signaling	0.0075	FOS, ATF3
Glucocorticoid receptor regulatory network	0.0077	FOS, EGR1
Sumoylation by RanBP2 regulates transcriptional repression	0.0174	RANBP2
JNK signaling in the CD4+ TCR pathway	0.0206	MAP2K4
Ras signaling in the CD4+ TCR pathway	0.0222	FOS
Hypoxic and oxygen homeostasis regulation of HIF-1-alpha	0.0284	TCEB2
Cellular roles of Anthrax toxin	0.0346	MAP2K4
VEGFR3 signaling in lymphatic endothelium	0.0361	MAP2K4
S1P2 pathway	0.0377	FOS
PDGFR-alpha signaling pathway	0.0377	FOS
ALK1 signaling events	0.0392	ACVR2A
Signaling events mediated by PRL	0.0392	EGR1
TRAIL signaling pathway	0.0438	MAP2K4
Regulation of CDC42 activity	0.0453	APC
S1P3 pathway	0.0453	CXCR4
CD40/CD40L signaling	0.0469	MAP2K4
Canonical Wnt signaling pathway	0.0469	APC
p38 MAPK signaling pathway	0.0469	TXN
Calcium signaling in the CD4+ TCR pathway	0.0484	FOS
Nongenotropic Androgen signaling	0.0484	FOS
Nephrin/Neph1 signaling in the kidney podocyte	0.0499	MAP2K4
IL12 signaling mediated by STAT4	0.0499	FOS

## Conclusions

In this work, a filter-based feature selection method, biomarker identifier (BMI), has been applied to find potential biomarkers for lung cancer from microarray data. BMI measures the potential value of each gene as a biomarker candidate by combining various statistical measures to assess its ability to distinguish between two data groups of interest. We evaluated BMI performance on two public microarray data sets: one from the MicroArray Quality Control project and the other from smokers with and without lung cancer. BMI was compared with other popular filter-based feature selection methods on both data set and showed competitive performance in selecting useful features for various classification algorithms. Since of the latter data set includes information regarding specific genes whose tissue differentiation relevance has been validated by quantitative RT-PCR, we also compared how these genes were ranked by different feature selection algorithm. The validated genes generally were assigned higher ranks by BMI than by other methods, implying that BMI should be effective in identifying biomarkers that show differential expression in cancerous samples. We also compared BMI with the approach in the original analysis conducted on the lung cancer microarray data [13] by contrasting the classification performance using selected genes from each method. Given models trained for various classification algorithms, classifiers based on genes selected by BMI showed better performance than those from original study. Finally, in evaluating whether the genes selected by BMI have known biological function related to (lung) cancer, we analyzed their pathway disposition and found that many genes were associated with known cancer-related pathways. Thus we can conclude that BMI is a suitable technique for phenotypic classification of microarray data and may provide a reasonable mechanism for identifying viable diagnostic biomarker candidates. Based on the results in this study, we are pursuing a follow-up study using BMI to identify biomarkers suitable for the lung cancer analysis with experimental data on clinically derived tissues.

## Competing interests

The authors declare that they have no competing interests.

## Authors' contributions

IL participated in the design of the study, performed the statistical analysis and drafted the manuscript. GL and MV conceived of the study, and participated in its design and coordination. All authors read and approved the final manuscript.
